# Big data are coming to psychiatry: a general introduction

**DOI:** 10.1186/s40345-015-0038-9

**Published:** 2015-09-29

**Authors:** Scott Monteith, Tasha Glenn, John Geddes, Michael Bauer

**Affiliations:** Michigan State University College of Human Medicine, Traverse City Campus, 1400 Medical Campus Drive, Traverse City, MI 49684 USA; ChronoRecord Association, Inc., Fullerton, CA 92834 USA; Department of Psychiatry, University of Oxford, Warneford Hospital, Oxford, OX3 7JX UK; Department of Psychiatry and Psychotherapy, Universitätsklinikum Carl Gustav Carus, Technische Universität Dresden, Fetscherstr. 74, 01307 Dresden, Germany

## Abstract

Big data are coming to the study of bipolar disorder and all of psychiatry. Data are coming from providers and payers (including EMR, imaging, insurance claims and pharmacy data), from omics (genomic, proteomic, and metabolomic data), and from patients and non-providers (data from smart phone and Internet activities, sensors and monitoring tools). Analysis of the big data will provide unprecedented opportunities for exploration, descriptive observation, hypothesis generation, and prediction, and the results of big data studies will be incorporated into clinical practice. Technical challenges remain in the quality, analysis and management of big data. This paper discusses some of the fundamental opportunities and challenges of big data for psychiatry.

## Introduction

Digital data are collected at an incredible rate. With 2.5 quintillion (2.5 × 10^18^) bytes of data generated every day, 90 % of the world’s data were created in the past 2 years (IBM 2015). This is due in part to the revolutionary belief that data and the unexpected information it contains are valuable (Economist 2010; MIT Sloan and IBM 2010; Hill 2013). Data as a critically important source of knowledge, insights and value are transforming every aspect of our world, including healthcare. There are now many successful standalone businesses that sell data, analytic tools and data analysis (ATKearney 2013). The market that is currently referred to as big data, including hardware, software and services was estimated at about $19 billion in 2013 (Kelly 2014). Healthcare is one of the fastest growing segments of the digital world, with healthcare data increasing at a rate of about 50 % per year (IDC 2014). There are three primary sources of big data in healthcare: providers and payers (including EMR, imaging, insurance claims and pharmacy data), omic data (including genomic, epigenomic, proteomic, and metabolomic data) (Starren et al. 2013), and patients and non-providers (including data from smart phone and Internet activities, sensors and monitoring tools) (Glenn and Monteith 2014).

The growth of big data in psychiatry will provide unprecedented opportunities for exploration, descriptive observation, hypothesis generation, and prediction for clinical, research and business issues. The results of big data analyses will be incorporated into standards and guidelines and will directly impact clinical decision making. Psychiatrists will increasingly have to evaluate results from research studies and commercial analytical products that are based on big data. In addition to the opportunities, multiple challenges remain relating to data quality, acquisition and processing, analytical methodology and interpretation. The purpose of this article is to discuss some of the fundamental features of big data that will be a part of psychiatry in the near future. The wide variety of ethical issues related to big data in society including individual privacy, informed consent, reuse of data, involvement of commercial organizations, and attitudes towards the boundaries between public and private are outside the scope of this article.

## What is big in big data?

There are many definitions of big data and the differences in perspective reflect the broad impact big data are having on modern life. The most common definition describes characteristics of big data as volume, velocity and variety (Laney 2001). Volume refers to the massive size of big datasets. A typical 500-bed hospital contains more than 50 petabytes (50 × 10^15^) of data (IDC 2014). An estimate of per patient data generated in an EMR is about 80 MB per year with 95 % of this being imaging data (Halamka 2011). Genomic data require 50 times more storage per patient than imaging data (Starren et al. 2013). With an estimated 1.2 billion ambulatory care visits in the US in 2014 (CDC 2014), and 78 % of physicians and 59 % of hospitals now using an EMR system (HHS 2014), the size of medical datasets will expand rapidly. The size of medical data refers not only to newly created data, but also to information that was generated in the past.

Velocity refers to the rate at which data are generated and must be acted upon, such as filtered, reduced, transferred and analyzed, as opposed to stored for future processing. As an extreme example, the Large Hadron Collider at the Center for European Nuclear Research (CERN) generates about 1 PB of raw data per second, of which one out of 10,000 events are passed through to processor cores where 1 % of the remaining events are selected for analysis (CERN 2015). In the commercial world, the proliferation of digital devices such as smartphones with applications that record locations, preferences, etc., using sensors and RFID tags has led to an unprecedented rate of data creation. Behavioral analytics for targeted advertising creates a need to process huge amounts of streaming data at very high rates of speed in near real-time for timely delivery of ads. Variety refers to the diverse data forms in big data, including structured (tabular such as in a spreadsheet or relational database), unstructured (such as text, imaging, video, and audio), and semi-structured (such as XML documents). About 80 % of the data in healthcare are unstructured (IBM 2013).

Big data is also defined by its complexity. In a traditional healthcare dataset, such as for a clinical trial, there are a large number of subjects (*n*) in comparison to a limited number of parameters (*p*) for each subject, referred to as a “large *n*, small *p*” problem (Spiegelhalter 2014; Sinha et al. 2009). Big data can expand this to where the number of subjects is extremely large in relation to the number of parameters. Big data may also change the fundamental relationship to a “large *n*, large *p*” problem where datasets not only have a very large number of subjects, but a very large number of parameters for each subject. Additionally, some data, such as from genomic microarray or fMRI, create “small *n*, large *p*” problems where there may be a huge number of parameters for a limited number of subjects (Spiegelhalter 2014; Fan et al. 2014). Both “large *n*” and “large *p*” problems create new and difficult computational and statistical challenges for analysis and interpretation of big data (Fan et al. 2014). Big datasets may also combine disparate datasets of very different dimensions. Bigness can be defined as data so multidimensional and complex that it must be reduced before it can be analyzed (Patty and Penn 2015), or as when current technology and methods (throughput and analytics) cannot provide timely and quality answers to data-driven questions (Kraska 2013; Jacobs 2009). The *n* and *p* characteristics of the datasets used in psychiatry research will have great impact.

Another perspective is that big data is defined by its impact on human sensemaking, where sensemaking is defined as the process used to analyze data and make decisions (Rohrer et al. 2014). Big data is too massive for humans to comprehend without the assistance of computer models (Weinberger 2012). The emerging field of visual analytics attempts to combine the data processing power of a computer with the outstanding human ability to recognize visual patterns (Ware 2012; Wong et al. 2012). Visual analytics systems use interactive visual interfaces to facilitate human analytical reasoning (Wong et al. 2012; Rohrer et al. 2014). While arising in the intelligence industry (Kielman et al. 2009; Rohrer et al. 2014), projects with this approach are being developed in biology and healthcare (Shneiderman et al. 2013; O’Donoghue et al. 2010).

Finally, bigness can be defined in the relation to changing attitudes to technology. With the new primacy of data, technologies are designed around the data instead of data being designed around the technologies (Gallagher 2013). The traditional role of IT within an organization including healthcare, of automating business processes, will have to change focus to handle data-intensive analytical processing, and make information more readily available to all (Kouzes et al. 2009).

## Other unique features of big data

Most data currently used in medical research, such as a randomized controlled trial, were designed and collected to answer a specific question. By contrast, a big dataset is designed to be re-used for many purposes, and to answer multiple questions including questions that cannot be anticipated at the time of data collection. Big data are often collected for reasons unrelated to research, such as an EMR, and multiple researchers are generally contributing data. The data may be physically stored in a distributed fashion across the globe. Big data are often combined with open (public access) data now available from governments worldwide, including a wide range of economic, health and climate data, and vital statistics. Furthermore, vast amounts of data available from commercial for profit companies will increasingly be involved in medical research. Big data ownership is fragmented across all the sources of data, including providers, payers, pharmaceuticals, governments, data brokers, technology providers and patients (Szlezák et al. 2014).

Unlike with smaller data projects, big data projects require the collaboration of people with diverse areas of expertise including physicians, biologists, statisticians, software engineers and developers, mechanical engineers, and network security analysts. The big data projects are often expensive to administer, and require detailed project management with procedures and quality standards for every aspect of dealing with data. Lessons learned in prior implementations of data projects such as NIH/NCI Cancer Biomedical Informatics Grid (caBIG) and the genomics project ENCODE may be of interest (NCI 2011; Birney 2012).

## Big data in general medicine and psychiatry

Big data provide many opportunities for scientific exploration. Clinical data mining can be used to answer questions that cannot be addressed with randomized clinical trials (Murdoch and Detsky 2013). For example, active postmarketing drug surveillance can use data from EMR, event reporting systems and social media (Moses et al. 2013; Harpaz et al. 2012). Other examples include situations where randomized clinical trials would be unethical such as in critical care, or where multiyear results are desired (Cooke and Iwashyna 2013). Big data also can help to determine whether conclusions derived from narrowly selected samples for randomized clinical trials are generalizable to a broader population (Murdoch and Detsky 2013). Big data allows new clinical questions to be asked and phenomena explored that were previously unavailable. Thus, observational data can be used to generate new hypotheses that may be more generalizable, and may help to create better randomized controlled trials (Titiunik 2015; Cooke and Iwashyna 2013). Randomized registry trials are being created, which randomize based on observational database information, and then integrate investigation with routine clinical care (Lauer and D’Agostino 2013; March et al. 2005).

Big data may allow the study of rare events. This includes the exploration of the relation between parameters such as genetic findings and rare diseases (Fan et al. 2014), and the study of those in the tails of distributions such as the small percent of the population with the highest healthcare expenditures (Cohen 2012). Large scale claims utilization databases based on data from community settings will be useful in epidemiologic research (Schneeweiss and Avorn 2005). Finally, observational data allow measurement of various parameters of real-world clinical practice.

Big data are already impacting every aspect of medicine. The secondary use of data has contributed to understanding variation in critical care treatment, including racial/ethnic and insurance-based disparities (Cooke and Iwashyna 2013). Other diverse examples of ongoing projects include whole slide images in pathology (Wilbur 2014), EMR surveillance for post-operative complications (FitzHenry et al. 2013), critical care databases for continual learning in the ICU (Celi et al. 2013), large clinical networks for outcomes research such as PCORnet (Collins et al. 2014) and the million veteran program (VA 2015), a new drug surveillance database from the FDA (2014), and using omics data to better understand immunity and vaccination (Nakaya et al. 2011).

Big data are also transforming psychiatry. Table 1 illustrates the potential impact of big data with examples of a wide range of recent projects. Observational evidence may be particularly important to psychiatry as the evidence available from randomized controlled trials may be incomplete, inconclusive or unavailable for many everyday clinical decisions (Bhugra et al. 2011). Furthermore, many patients who participate in clinical trials in psychiatry, including for bipolar disorder and schizophrenia, are not typical of those seen clinical practice (Zarin et al. 2005; Hoertel et al. 2013). Big data may help to create new clinical distinctions and phenotypes based on aggregated measurements of observational data (Altman and Ashley 2015; Hripcsak and Albers 2013). These new phenotypes may increase understanding of the heterogeneity present in psychiatric diagnoses such as bipolar disorder, and of the complex underlying genetics (Castro et al. 2015; Potash 2015). Big data may provide sufficient data to study subpopulations that are underrepresented in traditional samples, such as heroin addicts, using techniques such as integrative data analysis that combine independent data sets to product adequate sample sizes (Srinivasan et al. 2015; Curran and Hussong 2009). The maturing infrastructure to acquire, transmit, store and analyze exabyte-scale quantities of multisite neuroimaging data will expand knowledge of fundamental brain processes throughout normal life as well as in diseased states (van Horn and Toga 2014).

**Table 1:** Examples of a wide variety of projects using big data in psychiatry

Description	Primary finding	Number of subjects (*n*)	Data source	References
Create actuarial suicide risk algorithm to predict suicide in the 12 months after inpatient hospitalization for psychiatric disorder	52.9 % of posthospitalization suicides occurred after the 5 % of hospitalizations with the highest predicted suicide risk	40,820 soldiers hospitalized for psychiatric disorders. 421 predictors	38 army and DOD administrative data	Kessler et al. (2015)
Explore prevalence of substance use disorders (SUD) among psychiatric patients in large university system	24.9 % of patients had SUD; SUD associated with more inpatient and emergency care	40,999 psychiatric patients aged 18–64 years who sought treatment between 2000 and 2010	EMR-based psychiatry registry	Wu et al. (2013)
Ongoing study of cognitive impairment using neuroimaging and genetics	Neuroimaging phenotypes were significantly associated with progression of dementia	808 patients over age 65, including 200 with Alzheimer’s disease	20 derived neuroimaging markers plus 20 SNPs	Weiner et al. (2012)
Examine use of psychotropic drugs by patients without psychiatric diagnosis	58 % of those prescribed a psychiatric medication in 2009 had no psychiatric diagnosis	5,132,789 individuals who received prescription for psychotropic medication	Private medication claims database	Wiechers et al. (2013)
Analyze prescribing of psychotropic drugs by specialty	59 % written by general practitioners, 23 % by psychiatrists, 17 % by other physicians and providers	472 million prescriptions for psychotropic drugs	IMS database of 70 % of US retail pharmacy transactions for 2006–2007	Mark et al. (2009)
Compare risk of dementia in those 55 or older having traumatic (TBI) brain injury versus non-TBI trauma (NTT)	TBI increased risk for dementia over NTT	51,799 patients with trauma, of which 31.5 % had TBI	CA statewide administrative health database of ER and inpatient visits	Gardner et al. (2014)
Use machine learning to predict suicidal behavior text in EMR	Model obtained high specificity but low sensitivity, with PPV of 41 %	250,000 US veterans of Gulf War	Clinical records	Ben-Ari and Hammond (1991)
Investigate association between maternal and paternal age and risk of autism	Both increasing maternal age and increasing paternal age were independently associated with increased risk of autism	7,550,026 single births in CA 1989–2002. 23,311 with autism	Developmental services administrative data, birth certificate data	Grether et al. (2009)
Use natural language processing (NLP) to classify current mood state to identify treatment resistant depression	NLP models better than those relying on billing data alone	127,504 patients with diagnosis of major depression	EMR and billing data from outpatient psychiatry practices affiliated with large hospital	Perlis et al. (2012)
Analyze impact of Medicaid prior authorization for atypical antipsychotics on prevalence of schizophrenia among prison inmates	Prior authorization associated with greater prevalence of mental illness in inmates	16,844 inmates	Nationally representative sample from Census Bureau	Goldman et al. (2014)
Investigate incidence of severe psychiatric disorders following hospital contact for head injury	Increased risk of schizophrenia, depression, bipolar disorder and organic mental disorders following head injuries	113,906 people who had suffered head injuries, and were born between 1977 and 2000	Danish psychiatric central register	Orlovska et al. (2014)
Integrate depression screening, prescription fulfillment and EMR to improve care in primary care (PC)	Integration improved diagnosis and management of depression in PC	61,464 patients in PC in 14 clinical organizations	EMR, plus 4900 PHQ-9 questionnaires, plus fulfillment data for 55 % of patients	Valuck et al. (2012)
Analyze if SSRI/SNRI use prior to admission to ICU increased mortality risk	Increased hospital morality among those in ICU taking SSRI/SNRI before admission	14,709 patients with 2471 taking SSRI/SNRI	Multiparameter Intelligent Monitoring in Intensive Care database (data from EMR)	Ghassemi et al. (2014)
Evaluate safety of antipsychotic (AP) medication use in nursing homes	Dose-dependent increased risks of serious medical events such as myocardial infarction, stroke, infection, hip fracture, within 180 days of initiating AP treatment	83,959 Medicaid eligible residents ≥age 65 who initiated AP use after nursing home admission	Medicare and Medicaid claims from 45 states	Huybrechts et al. (2012)
Evaluate use of EMR to assist with phenotyping in bipolar disorder (BP)	Semiautomated data mining of EHR may assist with phenotyping of patients and controls	52,235 patients with at least one diagnosis of BP or mania, spanning 20 years	EMR, billing and inpatient pharmacy data	Castro et al. (2015)

Big data have fundamentally changed the ability to analyze human behaviors and actions. Huge quantities of data are created as a by-product of the routine transactions of daily life from smart phone and Internet activities including social medial, sensors and monitoring tools (Glenn and Monteith 2014). These data tend to provide near real-time measures of behaviors, rather than attitudes or beliefs (Groves 2011), which are becoming increasingly predictable. Examples of prediction from social media and sensor data include human motility (De Domenico et al. 2013; Gonzalez et al. 2008), friendships (Eagle et al. 2009), personality (Youyou et al. 2015), and private traits such as sexual orientation and ethnicity (Kosinski et al. 2013). Big data may reveal behavior that was previously difficult to detect, including those that are deliberately hidden, and allow comparisons between more precise samples of interest (Monroe et al. 2015). Integration of behavioral data with provider and omics data may also lead to the detection of new biomarkers of psychiatric illness, including bipolar disorder (McIntyre et al. 2014).

## Quality issues with big data

Many issues impact the quality of big data. Data acquired from different sources are created with different levels of accuracy, precision and timeliness, and data not created for research may lack sufficient quality for research. Combining data items from different databases requires an assumption that the items are sufficiently similar that equivalence can be determined. It is difficult to keep relationships among data clear over time in large databases with many near match inputs (NSA 2014). Furthermore, the vast majority of data are unstructured. With structured data, almost every data field can be analyzed, missing data can be measured, and the ratio of information to data is very high. In contrast, with unstructured data, information must be detected from within a mountain of data (Groves 2011).

Neither EMR nor administrative/claims data were created for research purposes, and contain many quality issues that impede their use in research. These include highly variable accuracy (Hogan and Wagner 1997; Chan et al. 2010), substantial missing data and difficulty of differentiating missing from negative values (Wells et al. 2013), inconsistent use of medical terminology (Halamka 2014), redundant data in text (Cohen et al. 2013), varying levels of detail (Hersh et al. 2013), lack of completeness and fragmentation of medical record across providers (Bourgeois et al. 2010), impact of reimbursement policies on claims data (Overhage and Overhage 2013), inaccurate ICD codes (O’Malley et al. 2005), temporary truncations due to insurance coverage issues (Overhage and Overhage 2013), and variations in data over time due to changing federal requirements (Halamka 2014). EMR data are difficult to compare even when using the same vendor product or within the same organization (Chan et al. 2010). EMR data may also lack the required provenance (metadata to trace an exact history of the data contents and ownership) for use in research (Buneman et al. 2000).

Some data from commercial firms, such as Internet behavioral data, are created by proprietary algorithms. These algorithms are not validated publicly and may be modified at any time, such as to improve customer service, which can impact their use in longitudinal studies (Lazer et al. 2014). Data from social media may include measurement or self-presentation errors, such as the finding that half of adult Facebook users have more than 200 friends in their network (Smith 2014), and malicious errors with at least 67 million Facebook accounts either duplicate, malicious or otherwise ‘fake’ (Munson 2014). Errors can be created when data from diverse sources are combined. For example, the ways that floating-point numbers are stored on common software/hardware platforms and handled by compilers may exhibit subtle differences with respect to floating-point computations that may lead to serious errors in big data processing (Monniaux 2008).

The multidimensional complexity of big data requires that it is reduced before it can be practically analyzed, even using advanced tools. The more complex the data, the more reduction is done and the selection of which data should be retained versus which data discarded is crucial (Patty and Penn 2015). There are a wide range of methodologies for dimension reduction, with much active research in this field (Wolfe 2013). The selection of appropriate technique is related to the type of data involved, such that the process to extract information from imaging data is very different from that used to find information in unstructured text (Jagadish et al. 2014). Deciding which parameters are important is a subjective process, and may remove the natural variability that may challenge preconceived assumptions (Bollier et al. 2010). Furthermore, it is difficult to interpret context in big data as the sheer volume of data increases (Boyd and Crawford 2012). It can also be difficult to distinguish findings of interest from hardware and software errors, such as when filtering data from sensors (Jagadish et al. 2014). Data reduction methodologies are of particular importance to medicine since most secondary clinical databases contain only the data parameters of interest (Wang and Krishnan 2014).

## Analytical challenges for big data

Regardless of how big the data are, it is still a sample and must be representative of the population of interest. For example, although there is considerable interest in the analysis of Twitter content to monitor aspects of behavior such as suicide risk (Jashinsky et al. 2014) or the stigma of schizophrenia (Joseph et al. 2015), the Twitter user population is highly unrepresentative of the US population (Mislove et al. 2011). Conclusions based on social media apply only to the self-selected group who use the specific site. The demographic variables that limit the generalizability of social media include age, gender, ethnicity, income, geography and Internet skills (Mislove et al. 2011; Hargittai 2015). There are many other types of biases in big data (Ioannidis 2013), including in EMR and claims data (Kaplan et al. 2014). One type of bias in EMR and research databases may be underrepresentation of racial and ethnic minorities due to disparities in mental health care in psychiatry, primary care and clinical research (Cook et al. 2014; Lagomasino et al. 2011; Yancey et al. 2006). Other examples of bias detected in EMR and claims data are listed in Table 2.

**Table 2:** Examples of bias errors in EMR and claims data

Study description	Issue	Errors found	Patient source	References
Examine relationship between illness severity and quantity of data in EMR	Data sufficiency	Setting minimal data requirements for inclusion in a study cohort created bias toward selection of sicker patients	EMR records from 10,000 patients who received anesthetic services	Rusanov et al. (2014)
Investigate patterns in lab tests for potential impact on use in modeling EMR data	Context for interpreting lab tests results	Frequency of lab tests confounded by scheduled visits, such as every 3 months	EMR records from 14,141 patients	Pivovarov et al. (2014)
Repeat prior study of pneumonia severity index to demonstrate bias in EMR retrospective research	(a) Diagnostic consistency	Adding constraints to improve consistency of diagnostic cohort significantly changed the sample (decreased the size)	EMR records from 46,642 patients with indication of pneumonia	Hripcsak et al. (2011)
	(b) Small number of cases can have large impact on outcome	Very sick patients who die quickly in ER will not have symptoms entered into EMR, impacting mortality rates		
Investigate concordance of diagnosis of PTSD in EMR with diagnosis determined by SCID interview	Diagnostic accuracy	Over 25 % of EMR diagnoses in veterans were incorrect for PTSD. Those with least and most severe symptoms most likely to be accurate	Sample of 1649 veterans	Holowka et al. (2014)
Evaluate diagnosis of schizophrenia in EMR compared with chart review by psychiatrist	Diagnostic accuracy	Prevalence of schizophrenia was 14 % by coding, dropping to 1.8 % with manual review. Coding most accurate (74 %) for those with four or more coding labels	819 veterans in a pain clinic	Jasser et al. (2007)
Review whether written informed consent introduces selection bias in prospective observational studies using data from EMR	Written informed consent	Significant differences between participants and non-participants with inconsistent direction of effect	Review of 1650 citations. 17 studies included with 69 % of 161,604 eligible patients giving consent	Kho et al. (2009)
Analyze if underlying health of seniors impacts risk reduction for death and hospitalization associated with influenza vaccine	Selective prescribing of preventative measures	Greatest reduction in risk occurs before influenza season, indicating preferential receipt of vaccine by healthy seniors	72,527 people ≥65 years not residing in nursing homes, using plan administrative data	Jackson et al. (2006)
Investigate surprising protective effects attributed to preventative medications by examining association between statin use and motor vehicle and workplace accidents	Healthy-adherer bias (adherent patients more health seeking)	Statin users significantly less likely to be involved in motor vehicle and workplace accidents. Example of unmeasurable confounding in dataset	141,086 patients taking statins for prevention	Dormuth et al. (2009)
Passive case-finding for Alzheimer’s disease and dementia using medical records	Research center population not generalizable	Research center population younger, more severe disease, more educated than general population	5233 patients over age 70	Knopman et al. (2011)
Explore selection bias when comparing outcomes from cancer therapy using observational data in SEER database	Severity of illness, self-rated health, comorbidities	Improbable results. Adjustment techniques such as propensity scores insufficient. Some outcome measures caused by treatments	53,952 patients with prostate cancer in three therapy groups	Giordano et al. (2008)

Researchers commonly use big data to look for correlations yet the high dimensionality of big data creates analytical challenges. Classical statistical inference assumes that the explanatory variables included in a model and the resultant estimated errors are independent and uncorrelated. However, when statistical models are estimated that include a large number of explanatory variables these assumptions may be violated. The most common problems resulting from the presence of many variables are spurious correlations (many unrelated variables are correlated by chance), and incidental endogeneity (explanatory variables are correlated with the residual errors) (Fan et al. 2014). In addition, noise accumulation (the sum of estimating errors accumulated from many variables) may dominate the underlying signal and overwhelm the explanatory power of the model (Fan et al. 2014). New techniques are being developed to accommodate the issues unique to the analysis of high-dimensional data. However, if these issues are ignored and the assumptions of classical statistical inference are violated, the analytic results will likely be incorrect. As databases get larger, the potential for false findings grows exponentially (Spiegelhalter 2014). Other problems reported in the analysis of big data include overfitting of models, failure to establish stationarity in time series, and multiple comparison bias. Many results of big data analyses cannot be reproduced (Ince 2012; Ioannidis et al. 2009).

The widespread desire to use big data to go beyond correlation to determine causality presents additional analytical challenges. When trying to infer causality from observational healthcare data, confounding is a major problem due to the large number of potential parameters for each patient (Glass et al. 2013). There are a variety of approaches to adjust measured confounders to create comparison groups of patients with similar characteristics, such as propensity scores, stratification, matching, and regression (Austin 2011; Stuart 2010; Glass et al. 2013). These techniques may not address issues such as inconsistent or incorrect measurements, missing clinical variables, unknown or unmeasured confounders, and time-varying confounders and exposures (Glass et al. 2013; Polsky et al. 2009; Toh et al. 2011).

Statistically inferring causality using big data assumes all the needed variables are present, exactly the same problem as with small data (Titiunik 2015). If the parameters were incorrect in a small dataset, adding data will not solve the problem. Causal inferences require that important pretreatment parameters were not omitted and that posttreatment parameters were not included (Titiunik 2015). In the words of Hal Varian, chief economist at Google, “Observational data—no matter how big it is—can usually only measure correlation, not causality” (Varian 2014).

## Does big data replace small data?

There is a need for healthcare data of all sizes, and an important role remains for smaller data as well as big data. As the famous statistician John Tukey (1988) summarized data analysis “Neither exploratory nor confirmatory is adequate alone”. For example, smaller samples will continue to be used in randomized, clinical trials to determine drug efficacy for regulatory agencies, and to validate potential biomarkers (Ioannidis and Khoury 2013). Small to large samples with high-quality data will be used in observational studies, and can be combined with open data. Even commercial vendors such as Google create samples from big data based on criteria such as user names or geographic areas, and run randomly assigned treatment–control experiments to determine causality (Varian 2014). Smaller data are also easier to analyze, less expensive to manage, and can be effectively used by single institutions for many research purposes. However, with the increasing acceptance of remote patient monitoring, even small, clearly designed studies are beginning to generate big data. For example, daily self-reporting mood charting programs for bipolar disorder create large numbers of medication parameters (Bauer et al. 2013a; b). Other studies that prospectively capture streaming behavioral, neural and physiological data from a few hundred patients produce enormously complex, multidimensional time-stamped datasets.

It will become increasingly important in psychiatry to understand what size and type of database is most appropriate for the problem being addressed. Huge amounts of data collected for reasons that are unrelated and irrelevant to the question at hand may not be of value. However, as more precise analytics are available, big data will become increasingly useful for more types of questions. Continuing research will help to clarify which problems should be addressed with big data versus small data, which big data problems should be addressed by sampling, and which analytic techniques are most appropriate. Furthermore, as more hypotheses are generated from observational data, new procedures will be required to determine which hypotheses should be further investigated using randomized clinical trials (Drazen and Gelijns 2014).

In conclusion, data from clinical, administrative, imaging and omics, and the coming flood from patient Internet activities, sensors and monitoring tools will provide unprecedented opportunities for psychiatry. Despite many technical challenges, new approaches are rapidly being developed that will allow the use of big datasets to increase understanding of existing and new questions in psychiatry.
